# Exploring the Multi-Tissue Crosstalk Relevant to Insulin Resistance Through Network-Based Analysis

**DOI:** 10.3389/fendo.2021.756785

**Published:** 2022-01-18

**Authors:** Linlin Yang, Linquan Yang, Xing Wang, Hanying Xing, Hang Zhao, Yuling Xing, Fei Zhou, Chao Wang, Guangyao Song, Huijuan Ma

**Affiliations:** ^1^ Hebei Key Laboratory of Metabolic Diseases, Shijiazhuang, China; ^2^ Clinical Medical Research Center, Hebei General Hospital, Shijiazhuang, China; ^3^ Department of Endocrinology, Hebei General Hospital, Shijiazhuang, China; ^4^ Department of Internal Medicine, Hebei Medical University, Shijiazhuang, China

**Keywords:** network analysis, tissue crosstalk, multi-tissue analysis, glucose homeostasis, insulin sensitivity

## Abstract

Insulin resistance (IR) is a precursor event that occurs in multiple organs and underpins many metabolic disorders. However, due to the lack of effective means to systematically explore and interpret disease-related tissue crosstalk, the tissue communication mechanism in pathogenesis of IR has not been elucidated yet. To solve this issue, we profiled all proteins in white adipose tissue (WAT), liver, and skeletal muscle of a high fat diet induced IR mouse model *via* proteomics. A network-based approach was proposed to explore IR related tissue communications. The cross-tissue interface was constructed, in which the inter-tissue connections and also their up and downstream processes were particularly inspected. By functional quantification, liver was recognized as the only organ that can output abnormal carbohydrate metabolic signals, clearly highlighting its central role in regulation of glucose homeostasis. Especially, the CD36–PPAR axis in liver and WAT was identified and verified as a potential bridge that links cross-tissue signals with intracellular metabolism, thereby promoting the progression of IR through a PCK1-mediated lipotoxicity mechanism. The cross-tissue mechanism unraveled in this study not only provides novel insights into the pathogenesis of IR, but also is conducive to development of precision therapies against various IR associated diseases. With further improvement, our network-based cross-tissue analytic method would facilitate other disease-related tissue crosstalk study in the near future.

## Introduction

Insulin resistance (IR) is a precursor event to metabolic syndromes and underpins many metabolic disorders such as obesity, type 2 diabetes (T2D), and cardiovascular disease (1, 2). So far, considerable efforts have been devoted to investigate the pathogenesis of IR, thereby revealing several molecular basis for IR in individual tissues (3, 4). However, as a metabolic disorder affecting multiple organs, the tissue crosstalk mechanism underlying IR has not been elucidated yet.

In physiological conditions, tissues in body are not isolated and their dynamical interplay lays a cornerstone for the maintenance of whole-body homeostasis. Inter-tissue communication is such a vital mechanism for metabolic regulation (5, 6). Several lines of investigations even pointed a crucial role of tissue communication in metabolic diseases (7, 8). For example, by integrating multi-tissue transcriptomics data, the human metabolic activities in healthy and unhealthy conditions can be precisely predicted (9). By detecting metabolic profiles of eight tissues, circadian metabolic relationships were found to be rewired by nutrient challenges (10). Based on observations from multiple tissues, these outstanding works offered a whole-organism view for the understanding of metabolism. Nevertheless, due to the lack of effective means for crosstalk exploration, the tissue communications related to metabolic regulation were merely researched.

Although previous researchers tried to use the pair of secreted molecules and their receptors to describe inter-tissue connections (5, 11), there is still lack of a systematical characterization for the entire process of tissue crosstalk and related regulatory events. Network analysis is a powerful technology which helps to reproduce all activities within a biological system (12) and hence has been tried to solve multi-tissue problems (13). Theoretically, the tissue communications can be represented in form of the interface across networks of single tissue. Unfortunately, so far, most of the existing multi-network analysis algorithms were developed to mine either conservative motifs or dissimilarities among different networks (14–16). A method targeting interactions across networks, especially multi-organ networks, has not been reported yet.

To solve this issue, we proposed a network-based strategy to explore IR related tissue communications. The protein profiles of insulin sensitive organs, namely, white adipose tissue (WAT), liver, and skeletal muscle were detected in an insulin resistance mouse model *via* proteomics. A network-based method was developed to identify the interface between tissues, by which cross-tissue interactions as well as their up and downstream functions were particularly inspected. The functional interpretation of IR related tissue crosstalk not only provided a novel framework for exploring multilayer network connections, but also would benefit further precision therapies against various IR associated diseases.

## Methods

### Animals and Samples

Previous studies have shown strong causative relation between restricted intrauterine growth and adult metabolic reprograming in rodents (17, 18). To reduce the potential impact of uneven intrauterine nutrition accompanied by different litter size, we used a previously developed ICR mouse model (19). Adult (6- to 8-week-old) male and female ICR mice were purchased from Beijing Vital River Laboratory Animal Technology Co., Ltd. and caged. Three-week-old pups were weaned and randomly assigned to insulin resistance (IR) and control (Con) group. Respectively, they were fed *ad libitum* for 19 weeks with either high fat diet (n = 9) composed of 60% Kcal from fat (Beijing HFK Bioscience Co., LTD., H10060) to induce IR or standard chow diet (n = 12, Beijing HFK Bioscience Co., LTD., H10010). All mice were maintained on a 12 h-light/12 h-dark cycle in a specific pathogen-free barrier facility. Mice were fasted overnight before sacrifice. Liver, epididymal fat, quadriceps femoris and gastrocnemius were collected, shock-frozen in liquid nitrogen and stored at −80°C. All animal experiments were performed with the approval of the Animal Ethics Committee of Hebei General Hospital.

### Assessment of Insulin Sensitivity

To assess the insulin sensitivity of experimental mice, intraperitoneal glucose tolerance test (IPGTT) was performed. Mice were fasted for 8 h with free access to water before IPGTT. For each mouse, 50% glucose was injected in abdominal cavity at dose of 2 g glucose per kg body weight (2 g/kg BW). Blood glucose of fasting (0), 15, 30, 60, and 120 min were detected from tail vein blood by glucose analyzer (OneTouch^®^ UltraVue™). Fasting level of peripheral insulin was measured by ELISA or according to manufacturers’ instructions (80-INSMSU-E01, APLCO).

### TMT-Based Proteomics Analysis

A total of 9 subjects/group were selected for TMT-based quantitative proteomics. In this study, technical replicates were used to increase the number of identified peptides and also to improve the quantification. Equal protein extracts from each subject in the same group were pooled together and trypsin digested to perform LC–MS/MS analysis, which was repeated for three times. Details on sample preparation, LC–MS/MS assay and data analysis were provided in **Supplementary Methods**. The proteomics data has been deposited to PRIDE (20) with identifier PXD021046. Proteins with Fold Change ≥1.2 or ≤0.83 and P-value <0.05 (Student’s t test of log2 transformed signals) were regarded as differentially expressed proteins (DEPs) between two groups.

### Functional Enrichment Analysis

The Gene Ontology annotation about biological process of DEPs were obtained by BINGO (21). Pathway distribution of DEPs was extracted from KEGG (22). For better functional interpretation, disease pathways and global maps in KEGG were excluded and the remaining basic pathways were selected into subsequent enrichment analysis. False discovery rate (FDR) correction of hypergeometric test was used to measure the significance of differential proteins co-existing in a GO term or KEGG pathway. FDR <0.05 was regarded as a sign of significance.

### Identification of Metabolic Enzymes and Signaling Proteins

Metabolic enzymes and signaling proteins are two kinds of widely-studied functional proteins. In this study, we identified enzymes from the protein profiles based on whether there was an EC number corresponding to the protein. To exclude kinases which functioned as signaling proteins, metabolic enzymes were further restricted as enzymes that could only be mapped onto a metabolic pathway in KEGG. As many KEGG signaling pathways contain both signaling proteins and their target enzymes, we defined signaling proteins as those could be mapped to signaling pathways but not to metabolic ones. Chi-square test was used to assess the difference in proportions of enzyme and signal protein within each tissue’s protein profile.

### Constructing Tissue-Specific Networks of IR

Networks of liver, skeletal muscle, and WAT were separately constructed *via* a developed core network generating strategy (23). Firstly, a Perl script was written to extract all the proteins and their interactions from the *.kgml files of each mouse pathway in KEGG (22). Then, for each tissue, its DEPs were regarded as seed nodes and their connected proteins according to KEGG were screened. Afterwards, the seed nodes and their neighbors were connected to generate a seed net specific to one tissue. Finally, this seed net was simplified by Steiner minimal tree algorism, helping to cut unnecessary branches mainly composed by non-DEPs and keep important nodes bridging seed proteins (24). In order to avoid loss of information, all the connected components in the seed net were traversed during the Steiner tree step. In addition, to reduce the computational complexity, the constructed networks were all undirected. Finally, all trees obtained from the Steiner step were retained to constitute the tissue-specific network.

To assess the goodness of these tissue-specific networks, their topological structures, namely, connected nodes, edges, and density were compared to random situations. Here, density is defined as the ratio of existing edges (E) to all potential ones (E * (E − 1)/2), which descripts sparsity of a net. For each tissue, a protein set with the same node number as differential proteins was randomly selected from the background network, thereby generating a random network. In total, 100 random networks were constructed for each tissue-specific network. Compared to corresponding randomizations, tissue-specific network showing all parameters with |Z| >2.33 (25) was supposed to be successfully constructed.

### Constructing the Interface of Multi-Tissue Crosstalk Relevant to IR

The interface of tissue crosstalk was constructed to investigate the communications between different tissues involved in IR. As primary proteins responsible for tissue communication, secreted proteins were identified. Based on Uhlén’s prediction (26), human secreted proteins were converted to their homologous genes in mouse. Any proteins that cannot be identified as secreted ones were assigned into non-secreted proteins. Then, we used the manually curated information in KEGG PATHWAY (22) as background knowledge for regulatory relationships between proteins. Notably, these collected interactions were directed, which further helped to define the up and downstream processes involved by a given protein. For each secreted DEPs in tissue A, their regulated non-secretory proteins in tissue B were searched in network of tissue B and then regarded as non-secreted receptors. Thus, the cross-tissue interactions between secretory DEPs and their cross-tissue targets were extracted. Afterwards, to find the inner-tissue regulatory events relevant to tissue communication, the (a) connections between secreted DEPs and their upstream neighbor DEPs and (b) those between cross-tissue targets and their downstream neighbor DEPs within one organ were identified. The inter- and intra-tissue connections together constituted the interface of tissue crosstalk.

In addition, for each tissue, to exclude the interference of secreted proteins coming from other organ, their tissue specificity was further estimated according to the Genotype Tissue Expression (GTEx) Project (27). Among the three insulin sensitive tissues, proteins whose mRNA levels were more than 10 times lower than any other tissue were considered to be low-expressed and were excluded from this tissue when constructing the interface.

### Functional Analysis of Cross-Tissue Interface

To functionally interpret the interface of tissue crosstalk, the up and downstream regulatory processes of cross-tissue interactions were investigated. Here, the upstream processes were defined as the pathways regulating or interacting with secreted proteins. In the interface of crosstalk, upstream processes can be measured by the pathways enriched by upstream and secreted proteins within one tissue (FDR <0.05). Similarly, downstream processes were defined as the pathways receiving regulation from secreted proteins and can be measured by pathways enriched by non-secreted proteins and their targets on the interface.

### Validation

Since pooled samples were used during proteomics detection, which might implicate the significance of DEP signals, the expression of candidate DEPs was further validated by Western blotting (WB) assay of individual subjects. Total protein of each sample was extracted using total protein extraction kit (BC3710, Solarbio) according to the manufacturers’ instructions. Western blots were performed by using specific antibody of CD36 (ab133625, Abcam), ACTIN (4970, CST), fatty acid-binding protein 1 (FABP1, 13368, CST), fatty acid-binding protein 3 (FABP3, 10676-1-AP, Proteintech), fatty acid-binding protein 4 (FABP4, 3544, CST), peroxisome proliferators-activated receptor α (PPARα, 15540-1-AP, Proteintech), peroxisome proliferators-activated receptor γ (PPARγ, 2443, CST), and phosphoenolpyruvate carboxykinase 1 (PCK1, 12940, CST). The images were captured *via* Minichemi 610 Plus (Sagecreation, Beijing, China). Serum thrombospondin 4 (THBS4) were measured *via* enzyme-linked immunosorbent assay (ELISA) according to manufacturers’ instructions (SED824Mu, Cloud-Clone Corp.).

## Results

### High Fat Diet Induced Insulin Resistance

**Figure 1:**
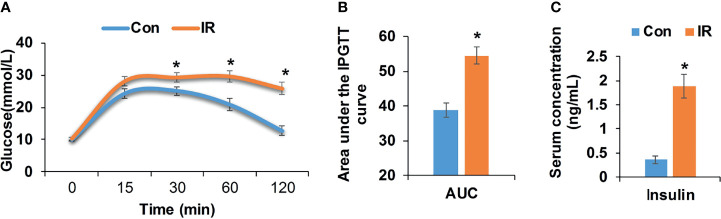
Metabolic measurements of experimental mice. **(A)** Intraperitoneal glucose tolerance test (IPGTT, 2 mg/kg), age 21 weeks (Con, n = 12; IR, n = 9); **(B)** Area under the IPGTT curve; **(C)** Fasting insulin level of experimental mice, age 21 weeks (n = 6/group). *P < 0.05, compared to control group, Student’s t-test; the normality of corresponding data was determined by Shapiro–Wilk test (P > 0.05).

Compared with control group, high fat diet fed mice showed significantly higher body weight and body mass index (BMI) (P <0.05, two-tailed Student’s t-test, **Supplementary Figures 1A, B**). After 17 weeks of feeding, high fat diet fed mice showed elevated blood glucose at 0.5, 1, and 2 h after injection of glucose (P <0.05, two-tailed Student’s t-test, **Figure 1A**). Correspondingly, the area under intraperitoneal glucose tolerance test (IPGTT) curve (AUC) was significantly enlarged as well (P <0.05, two-tailed Student’s t-test, **Figure 1B**). Besides, higher fasting insulin demonstrated hyperinsulinemia in high fat diet fed mice (P <0.05, two-tailed Student’s t-test, **Figure 1C**), further confirming insulin resistance in IR group.

### Overview of Tissue-Specific Protein Profiles

**Figure 2:**
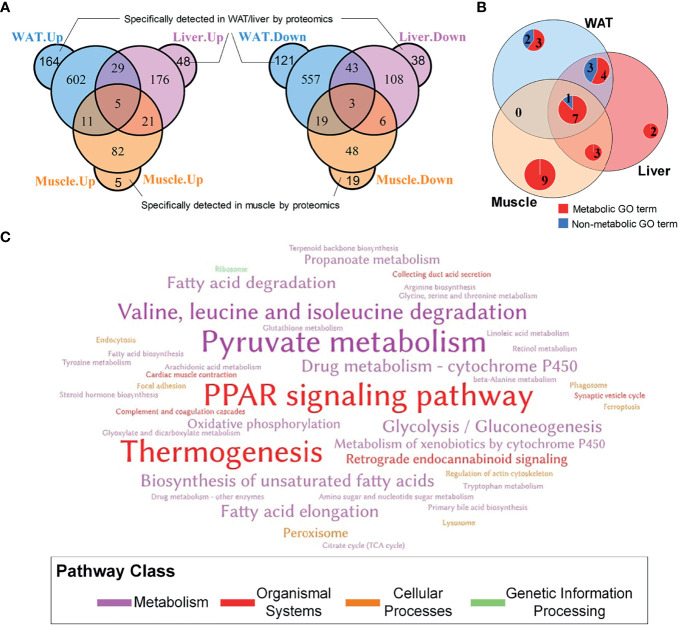
General information of DEPs in WAT, liver and skeletal muscle. **(A)** Overlapping of differential proteins; **(B)** Coincidence of TOP20 enriched GO terms, where the TOP20 GO terms with the lowest FDR in distinct tissue were graphed; **(C)** Word cloud of KEGG pathways enriched by tissue-specific protein profiles. The words with the largest font size represent pathways simultaneously enriched by three tissues; the words with medium font size represent pathways commonly enriched by two tissues; and the words with small font size represent pathways only enriched by one tissue.

In total, 5,854, 5,116, and 3,039 proteins were respectively quantified in WAT, liver, and skeletal muscle by proteomics. A total of 1,554, 477, and 219 differential proteins were identified in WAT, liver, and skeletal muscle, which are respectively listed in **Supplementary Tables 1–3**. It was unexpected that only 8 proteins were simultaneously up/downregulated in these tissues (**Figure 2A**), which are listed in **Supplementary Table 4**. Despite the low coincidence rate, the three DEP profiles showed quite similar biological functions. Among the TOP20 most enriched GO terms in the three tissue (see **Supplementary Figure 2**), there were more than 50% were annotated as metabolism-related processes (**Figure 2B**), which was in line with their mission as metabolic organs. Similarly, the three sets of DEPs commonly enriched in 3 KEGG pathways: pyruvate metabolism, PPAR signaling pathway and thermogenesis (**Figure 2C**), which accounted for 12.5, 9.4, and 21.4% of total pathways enriched by WAT, liver, and muscle. Notably, PPAR pathway was the only signaling pathway simultaneously enriched by the three tissues, highlighting its important role in regulation of IR.

### Tissue-Specific Networks of IR Showed Widespread Metabolic Alterations

**Figure 3:**
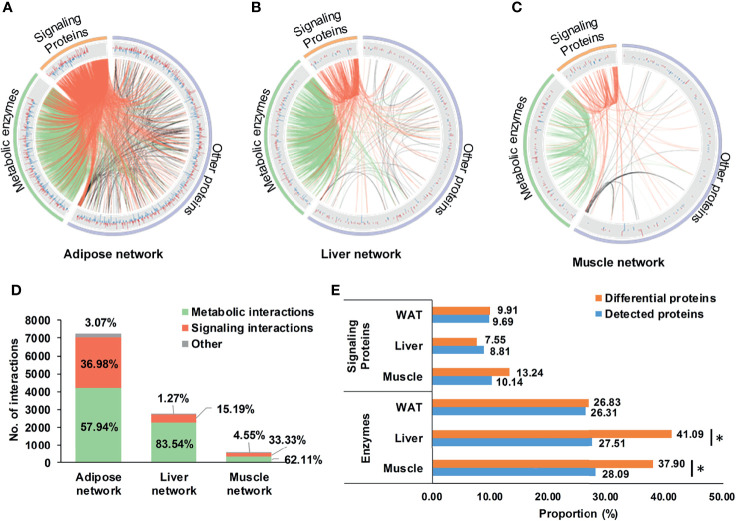
Tissue-specific networks of IR. **(A–C)** tissue-specific networks of IR. Where, fold changes of all differential proteins were shown in bar plots; signaling protein-related and metabolic enzyme-related interactions were respectively highlighted in red and green; **(D)** Distribution of metabolic and signaling interactions in tissue-specific networks; **(E)** Proportion of metabolic enzymes and signaling proteins in differential and detected proteins. *P < 0.05, chi-square test.

Based on DEPs, tissue-specific networks of WAT, liver, and skeletal muscle were constructed, which were shown in **Figures 3A–C**. Compared to corresponding random networks, significantly larger number of connected nodes, edges and density were found in adipose, liver, and muscle network (**Supplementary Figure 3**), suggesting successful construction of these nets.

It was worthy to note that enzyme-involved interactions dominated 57.9, 83.5, and 62.1% of the adipose, liver, and muscle network (**Figure 3D**), indicating widespread metabolic alterations in these tissues. This might be related to their proportion of metabolic enzymes. Separately, there were 26.8, 41.1, and 37.9% of DEPs in WAT, liver, and skeletal muscle belonging to metabolic enzymes, the proportion of which was consistently higher than signaling proteins (**Figure 3E**). Especially in liver and skeletal muscle, proportions of metabolic enzymes in DEPs were significantly larger than detecting pool (P <0.05, chi-square test, **Figure 3E**). It was strongly suggested that, rather than signaling interactions, wide-ranging metabolic processes were altered during the state of IR.

### Cross-Tissue Alterations Were Preferred in State of IR

**Figure 4:**
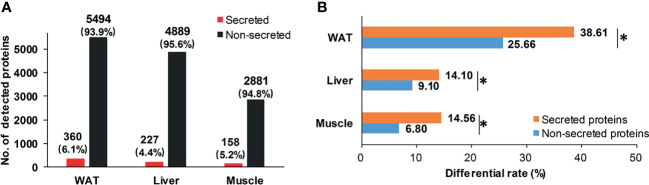
Distribution of secreted proteins. **(A)** Distribution of secretory and non-secretory proteins in detecting pool; **(B)** Differential rate of secretory and non-secretory proteins. *P < 0.05, chi-square test.

Considering secreted proteins are important functional vehicle for tissue communication, we screened secreted proteins in insulin sensitive tissues. According to Uhlén’s prediction (26), 360, 227, and 158 secreted proteins were identified in WAT, liver, and skeletal muscle (**Figure 4A**). The most secreted proteins were detected in WAT, implying its stronger potential for tissue communication than liver and skeletal muscle. Intriguingly, although secreted proteins in the three tissues only accounted for 5% of detected pool on average (**Figure 4A**), their differential rates were all significantly higher than non-secreted ones (P <0.05, chi-square test, **Figure 4B**). It was suggested that, compared to inner-tissue perturbations, the cross-tissue alterations may be prioritized in state of IR.

### The Cross-Tissue Interface of IR Was Featured by Metabolic Processes

**Figure 5:**
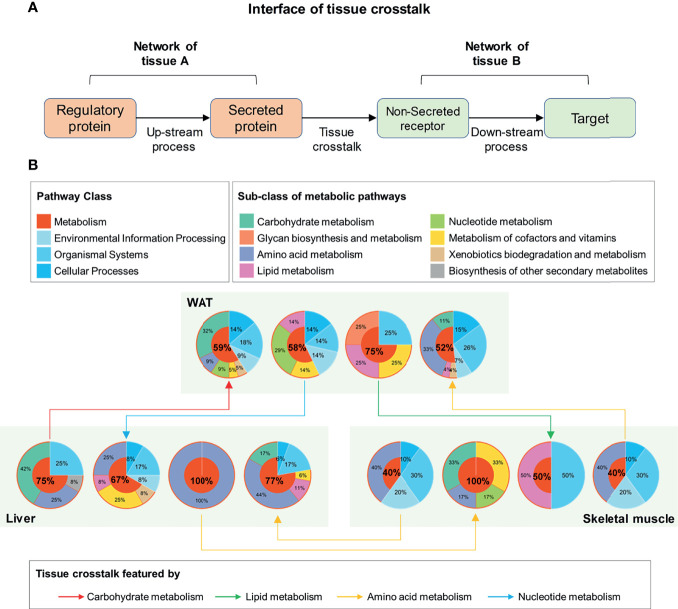
Functional interpretation of tissue crosstalk interface relevant to IR. **(A)** Schematic diagram of cross-tissue interface; **(B)** Functional classification of up and downstream processes in the interface of IR.

To explore tissue communications perturbated in process of IR, we used secreted proteins to trace the connection between networks of different tissues, the rationale of which was shown in **Figure 5A**. To guarantee the correct direction of tissue crosstalk, low-expressed secreted proteins in each tissue were firstly excluded. Then the cross-tissue interactions from secreted DEPs to non-secreted proteins were identified according to protein relationships annotated by KEGG database (22). Finally, the upstream DEP neighbors of secreted DEPs and the downstream DEP neighbors of non-secreted receptors were extracted to describe the up/down regulatory processes of tissue crosstalk. Together, the secreted DEPs, all possible cross-tissue receptors and their up/downstream DEP neighbors constituted the cross-tissue interface of IR. All involved interactions are listed in **Supplementary Table 5**.

Furthermore, the main function of these elements in the interface was investigated by searching their enriched pathways (FDR <0.05), which are shown in **Figure 5B**. Surprisingly, among the up- and down-stream processes (**Figure 5B**), metabolic pathways dominated an average of 66.08% of all enriched pathways (95% Confidence interval: 53.21–78.95%). This proportion was significantly higher than that in background database (P <0.01, one-sample z-test), where 91 (37.45%) out of 243 basic pathways were annotated as metabolic ones. It was suggested that the metabolism alterations accompanied by IR participated or even drove the communications between WAT, liver, and skeletal muscle. By characterizing the cross-tissue interactions *via* their dominant upstream pathways (**Figure 5B**), the crosstalk from liver to WAT was found as the only tissue communication driven by carbohydrate metabolism during IR. The main reason for this was due to the high proportion of glucose metabolic enzymes in upstream regulatory proteins of liver. Among the 6 differential upstream regulatory proteins in liver (**Supplementary Table 6**), 5 were enzymes related to glucose metabolism: glucosamine-6-phosphate isomerase, transketolase, glucose-6-phosphatase, hexokinase-3, and glucokinase. These enzymes may connect to secreted proteins through a series of linked metabolic reactions, thereby outputting abnormal glucose metabolic signals from liver. Besides, in WAT and skeletal muscle, carbohydrate metabolism accounted for one-third of the processes receiving hepatic signals (**Figure 5B**). It was stressed that liver played a pivotal role in regulation of glucose homeostasis during IR.

### CD36-PPAR Axis Was Highlighted as Vital Target for Tissue Crosstalk Relevant to IR

**Figure 6:**
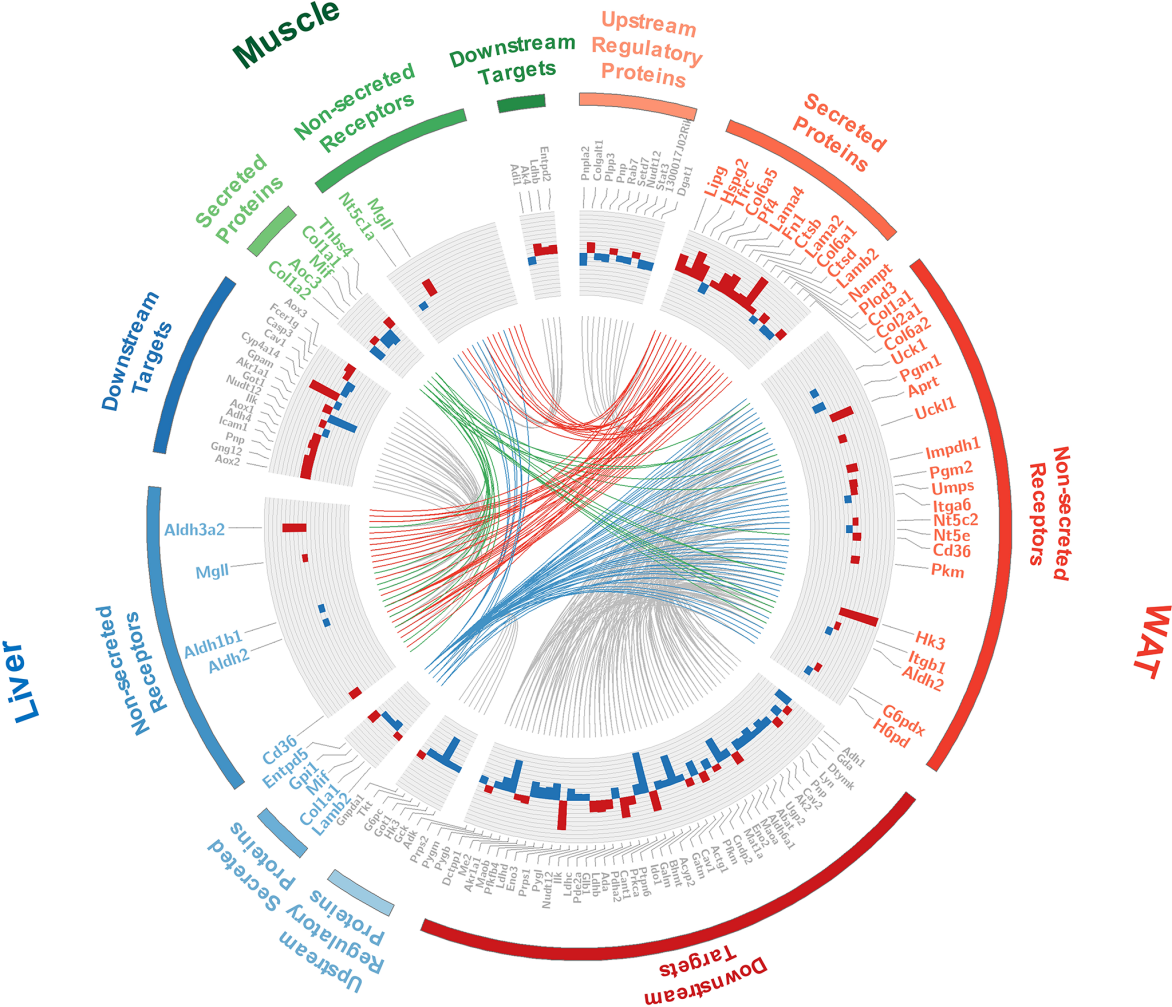
Detailed information of the tissue crosstalk interface relevant to IR. Where, fold changes of all differential proteins were shown in bar plots; the differentially expressed secretory proteins and their differential non-secreted receptors were labeled in color corresponding to each tissue; the up- and down-stream regulators were labeled in gray; the cross-tissue interactions started from WAT, liver, and skeletal muscle were respectively colored in red, blue and green.

**Figure 7:**
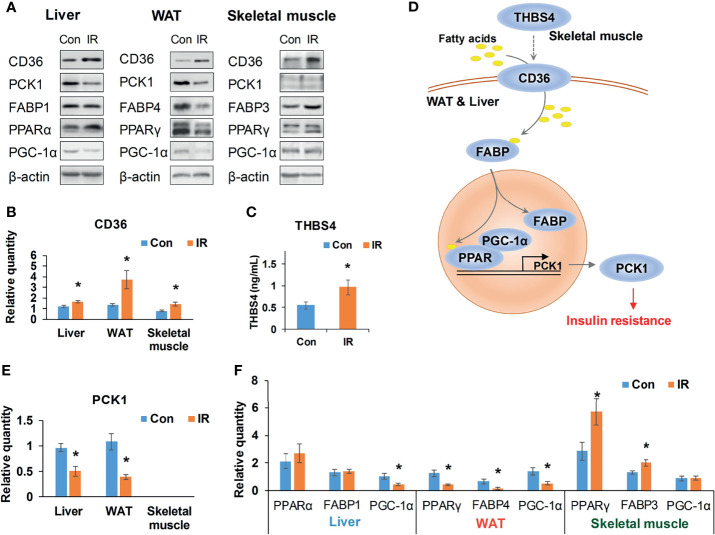
Validation of CD36–PPAR axis. **(A)** Representative WB bands of candidate proteins; **(B)** Relative quantity of CD36 based on WB bands (n =4/group); **(C)** Serum THBS4 level of experimental mice, (n = 9/group); **(D)** A potential cross-tissue mechanism of CD36-PPAR axis; **(E)** Relative quantity of PCK1 based on WB bands (n = 3–4); **(F)** Relative quantity of regulators in PPAR pathway based on WB bands (n = 4). *P < 0.05, compared to control group, Student’s t-test; the normality of corresponding data was determined by Shapiro–Wilk test (P > 0.05).

The interface of WAT, liver, and muscle is displayed in detail in **Figure 6**. As shown in **Figure 6**, many of the secreted proteins participating in tissue crosstalk were extracellular matrix (51.9%, see **Supplementary Table 7** for more details), while non-secreted receptors varied a lot. As one of the 5 commonly upregulated proteins in three tissues (**Supplementary Table 4**), CD36 was highlighted as receptor in WAT and liver. It was indicated that adipose and hepatic CD36 might be a vital receptor receiving cross-tissue signals. WB assay confirmed the elevated CD36 in three tissues (P <0.05, Student’s t-test, **Figures 7A, B**). Muscular CD36 was not included in the interface because no corresponding secreted proteins were differentially expressed in WAT and liver.

As it was exhibited in the cross-tissue interface of IR (**Figure 6**), both adipose and hepatic CD36 were linked with muscular thrombospondin-4 (THBS4). According to the proteomics detection, THBS4 in skeletal muscle was significantly increased in IR group (P <0.05, Student’s t-test, see **Supplementary Table 3**). Additionally, we also detected elevated THBS4 in serum of IR mice (P <0.05, Student’s t-test, **Figure 7C**). In liver and WAT, CD36-mediated lipid uptake could directly regulate the activity of PPAR pathway, which is an important controller for glucose homeostasis (**Figure 7D**). Hence, further effect of CD36–PPAR axis on metabolic regulation was subsequently evaluated. As a target gene of PPAR pathway, PCK1 is a rate-limiting enzyme of glyceroneogenesis in which gluconeogenic precursors such as pyruvate, lactate, and alanine were converted into the glycerol backbone of triglyceride (28, 29). It was identified as downregulated DEP in WAT and liver according to proteomics data. Following WB assay also confirmed decreased PCK1 in WAT and liver (P <0.05, Student’s t-test, **Figure 7E**), suggesting impaired metabolic homeostasis in IR group.

In addition, as the only signaling pathway commonly enriched by three tissues, the activity of PPAR pathway was examined. FABPs are a class of lipid carrier which binds and delivers ligands to PPAR. Unchanged FABP1 (P = 0.76, Student’s t-test) and significantly decreased FABP4 (P <0.05, Student’s t-test) was respectively detected in liver and WAT (**Figures 7A, F**), which was in line with the quantification result from proteomics. Although proteomics did not capture signals of PPAR, WB assay showed unchanged PPARα and decreased PPARγ in liver and WAT of IR group (**Figure 7F**). PGC-1α was consistently decreased in liver and WAT of IR group (P <0.05, Student’s t-test, **Figure 7F**). Therefore, PPAR pathway was suppressed in WAT and liver, which may resonate with the cross-tissue signals input by CD36. In parallel, the protein level of PPARγ and FABP3 in skeletal muscle was measured to be elevated in IR group (P <0.05, Student’s t-test, **Figure 7F**), suggesting enhanced muscular PPAR pathway. But neither proteomics nor WB assay detected protein expression of PCK1 in skeletal muscle, suggesting a rather weak effect of muscular PPAR pathway on PCK1 mediated metabolic dysfunction.

## Discussion

The present study developed a network-based strategy for tissue crosstalk identification, which was applied to the protein profiles of WAT, liver, and skeletal muscle in IR mice. From a network perspective, the tissue crosstalk relevant to IR was systematically explored and the potential cross-tissue mechanism was characterized.

As secretory proteins are essential for signal transduction between individual tissues, we screened them in all proteins detected from WAT, liver, and skeletal muscle. It was found that secreted proteins only accounted for a small fraction (5% on average) while presented significantly higher differential rates than non-secretory ones, suggesting a strong tendency of IR group towards impairing tissue communications. In this context, we proposed a secreted protein-based strategy for identifying tissue crosstalk relevant to IR. Inter-tissue connections including secretory proteins and their receptors as well as the inner-tissue up- and down-stream regulatory events were searched from distinct tissue-specific networks, thus generating a multi-tissue interface of IR.

We observed widespread metabolic alterations not only inside tissue-specific networks but also on the cross-tissue interface of IR, highlighting a significant role of metabolism in tissue communication during IR. Extraordinarily, liver was revealed as the only organ outputting abnormal glucose metabolic signals. Approximately 5 out of 6 differentially expressed upstream proteins of hepatic secreted proteins were glucose metabolism related enzymes. It was strongly suggested that, in IR subjects, the transduction of abnormal glucose metabolic signals was probably started from liver. As targets receiving hepatic signals, almost 1/3 downstream processes in WAT and skeletal muscle were classified into carbohydrate metabolism, further confirming the pivotal role of liver in regulation of glucose metabolism.

In the extracted cross-tissue interface of IR, we noticed that one of the 5 consistently upregulated proteins in three tissues CD36 was commonly highlighted as crosstalk receptors in both WAT and liver. In metabolic organs, CD36 is a major importer for fatty acids which were then delivered by FABPs to PPAR as ligands (30, 31). Coincidently, PPAR pathway was the only signaling pathway commonly enriched by three tissues, thus emphasizing the significance of CD36–PPAR axis in pathogenesis of IR. In fact, the effect of PPAR pathway in progressing IR was widely confirmed in individual tissues (32, 33), but its role in cross-tissue communications has not been clarified. As PPARs can activate the transcription of many rate-limiting enzymes and control glucose and lipid metabolism (34), we then respectively investigated the upstream cross-tissue signals and downstream metabolic effects of CD36–PPAR axis.

In IR group, both proteomics data and WB assay confirmed elevated CD36 in WAT, liver, and skeletal muscle, which was consistent with previous observations on individual tissues (35–38). As a membrane receptor, CD36 can bind various ligands like fatty acids and thrombospondins (39). In the crosstalk interface of IR, hepatic and adipose CD36 was pointed to receiving external signals from muscular THBS4. THBS4 is a member of thrombospondins which has been identified as secreted proteins regulating cell communications (40). We detected increased THBS4 in skeletal muscle and confirmed elevated THBS4 in serum of IR mice. Consistently, a recent study observed higher peripheral THBS4 levels in patients with type 2 diabetes (41). Conversely, THBS4^-/-^ mice also showed lower fasting glucose level than wild type and relieved hyperinsulinemia in nutrient challenge (42). It was indicated that the altered THBS4 highlighted in the upstream of CD36-PPAR axis may be important regulators for whole-body glucose metabolism.

To further verify the intracellular metabolic effect of CD36–PPAR axis, the protein expression of a PPAR target, PCK1 was examined. PCK1 is a key enzyme controlling the conversion of oxaloacetate into phosphoenolpyruvate, which is further used in glycerol 3-phosphate production for triglyceride synthesis (28, 29). In our study, both proteomics and WB assay detected decreased PCK1 in WAT and liver of IR mice. Low expression of PCK1 in liver and WAT has been implicated with IR through a lipotoxicity mechanism in transgenic mice (43). The reduced PCK1 in WAT can suppress fatty acids re-esterification and cause excessive accumulation of fatty acids derived toxic metabolites, thus resulting in lipotoxicity in ectopic tissues such as liver and skeletal muscle (44). Liver-specific PCK1-knockout experiment also confirmed the peripheral lipotoxicity of reduced hepatic PCK1 in chow-diet condition and its effect on exacerbating IR in high fat diet condition (45). Therefore, the downregulation of PCK1 here may contribute to the impaired glucose metabolism through a lipotoxicity mechanism, mechanically confirming a depressed effect of CD36–PPAR axis on glucose homeostasis in our IR mice.

Meanwhile, interference from the inherent activity of PPAR pathway was assessed by measuring main isotypes of its regulators, namely, FABPs, PPARs, and PGC-1α. FABPs are lipid carriers which bind and deliver ligands to PPAR (46). Our study respectively confirmed unchanged FABP1 and significantly reduced FABP4 in liver and WAT of IR group by proteomics and WB assay. Although proteomics did not capture signals of PPARs and PGC-1α, WB assay showed decreased PPARγ in both liver and WAT and downregulated PGC-1α in WAT of IR group. It was suggested that the activity of PPAR pathway was consistently depressed in liver and WAT of IR group. In parallel, skeletal muscle of IR group showed elevated FABP3 and PPARγ. But no signals of muscular PCK1 were captured by proteomics or WB, indicating a mechanism different from liver and WAT in regulation of glucose homeostasis. Taken together, it can be concluded that adipose and hepatic PPAR pathways are integral channels bridging external signals to intracellular glucose homeostasis in progression of IR.

In addition to the CD36-mediated signals, many extracellular matrix (ECM) related proteins were highlighted in the cross-tissue interface. ECM-related proteins are known as structural support can be released into circulation. A growing body of reports have shown strong correlation between altered ECMs and metabolic disorders (47, 48). Mechanically, ECM attachment has been reported to be required for cell communication (49) and regulation of metabolic processes (50), yet few direct pieces of evidence linking ECMs and tissue crosstalk can be found. In this study, our unbiased proteomics analysis provided a computational clue for the potential role of ECM-related proteins in tissue communication during IR, which may hint an alternative aspect of ECM functions.

This study is not without limitations. Our observation is based on static experiments in freshly isolated mouse organs. Dynamic experiments with further technical supports from conditional knockout animals or *in vivo* tracer techniques would provide more solid evidences for the transduction of cross-tissue signals. As obesity is the most common cause of IR (51), we chose high fat diet fed mice as our subjects. Inevitably, the results here may be specific to dietary fat induced IR. Technically, we used the same database during network construction and functional assessment and performed one sample z-test to reduce the possible circularity. Future random network analysis might be helpful to further improve this issue. Currently, as there was no standard dataset collecting tissue crosstalk interactions, we were not able to evaluate the performance of our approach. Alternatively, the results obtained from network analysis were partially verified by experimental assays. Overall, our systematical analysis of multi-organ proteomics paved a novel way to deciphering inter-tissue communications in dietary fat induced IR.

## Conclusion

It has been widely accepted that tissue communication underlies pathological basis of many diseases such as obesity, diabetes, metabolic syndromes, and so on. However, due to the lack of effective means to explore and functionally infer cross-tissue interactions, current study on disease-related tissue crosstalk has been tremendously hindered. To solve this issue, we proposed a network-based approach to explore the interface between multiple tissues, which was then applied to the proteomics data of WAT, liver, and skeletal muscle in IR mice. Interestingly, widespread metabolic alterations not only were observed in tissue-specific networks, but also dominated the cross-tissue interface related to IR, emphasizing their significance in development of IR. By quantifying functional pathways involved in the interface, liver was recognized as the only organ that can output abnormal carbohydrate metabolic signals, clearly highlighting its central role for glucose homeostasis. Especially, CD36–PPAR axis was identified and verified to potentially link inter-tissue signals with intracellular metabolism in liver and WAT, thereby promoting the progression of dietary fat induced IR through a PCK1-mediated lipotoxicity mechanism.

Although this CD36–PPAR axis mediated crosstalk mechanism requires validation from more functional experiments such as conditional transgenic assays, the present study still provides novel insights into understanding the tissue communications in pathogenesis of IR. With further improvement, our network-based function mining method would shed a light on other disease-related tissue crosstalk exploration in the near future.

## Data Availability Statement

The datasets presented in this study can be found in online repositories. The names of the repository/repositories and accession number(s) can be found below: https://www.ebi.ac.uk/pride/archive/, PXD021046.

## Ethics Statement

The animal study was reviewed and approved by the Animal Ethics Committee of Hebei General Hospital.

## Author Contributions

GS and HM conceived this research and designed the analysis. LinlY researched the data and prepared the primary manuscript. LinqY, XW, HX, HZ, YX, FZ, and CW performed the sample preparation and contributed to discussion. GS and HM are the guarantors of this work and, as such, had full access to all the data in the study and takes responsibility for the integrity of the data and the accuracy of the data analysis. All authors contributed to the article and approved the submitted version.

## Funding

This research was supported by grants from the National Natural Science Foundation of Hebei, China (C2019307081 to LinlY, H2019307108 to HM) and the Government Funded Program for Clinical Medicine Talent Training and Basic Research Project of Hebei, China (2015 to HM, 2017 to LinlY and HX).

## Conflict of Interest

The authors declare that the research was conducted in the absence of any commercial or financial relationships that could be construed as a potential conflict of interest.

## Publisher’s Note

All claims expressed in this article are solely those of the authors and do not necessarily represent those of their affiliated organizations, or those of the publisher, the editors and the reviewers. Any product that may be evaluated in this article, or claim that may be made by its manufacturer, is not guaranteed or endorsed by the publisher.
