# Supplementary material for: Exploring the Multi-Tissue Crosstalk Relevant to Insulin Resistance Through Network-Based Analysis
Source: Front Endocrinol (Lausanne). 2022 Jan 18;12:756785. doi: 10.3389/fendo.2021.756785 (PMC8805208; doi:10.3389/fendo.2021.756785)
Supplement: Supplementary file 1 [file DataSheet_1.zip › Supplementary Figures.DOCX]

Supplementary Figures


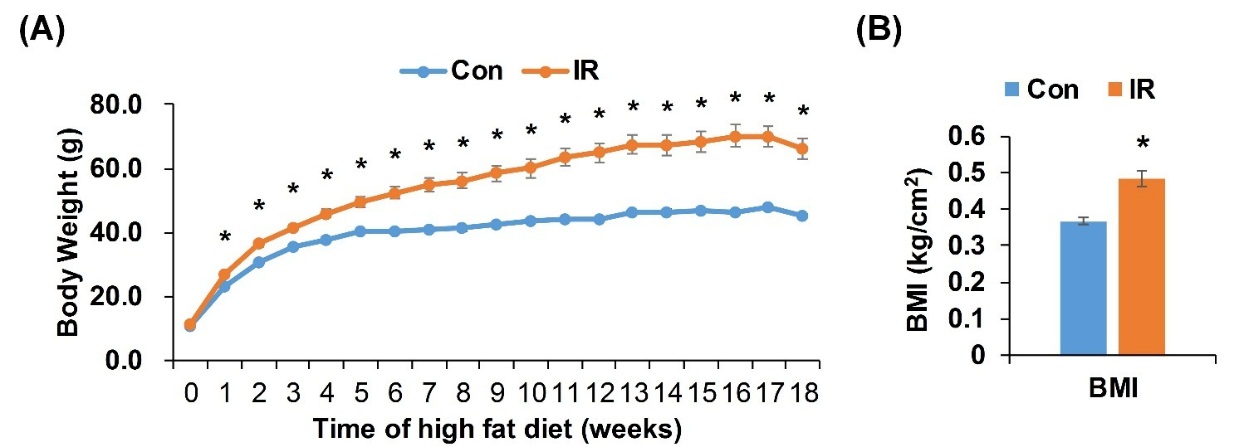


**Supplementary Figure 1. Body weight and BMI of experimental subjects.** age 21 weeks (Con, n = 12; IR, n = 9).


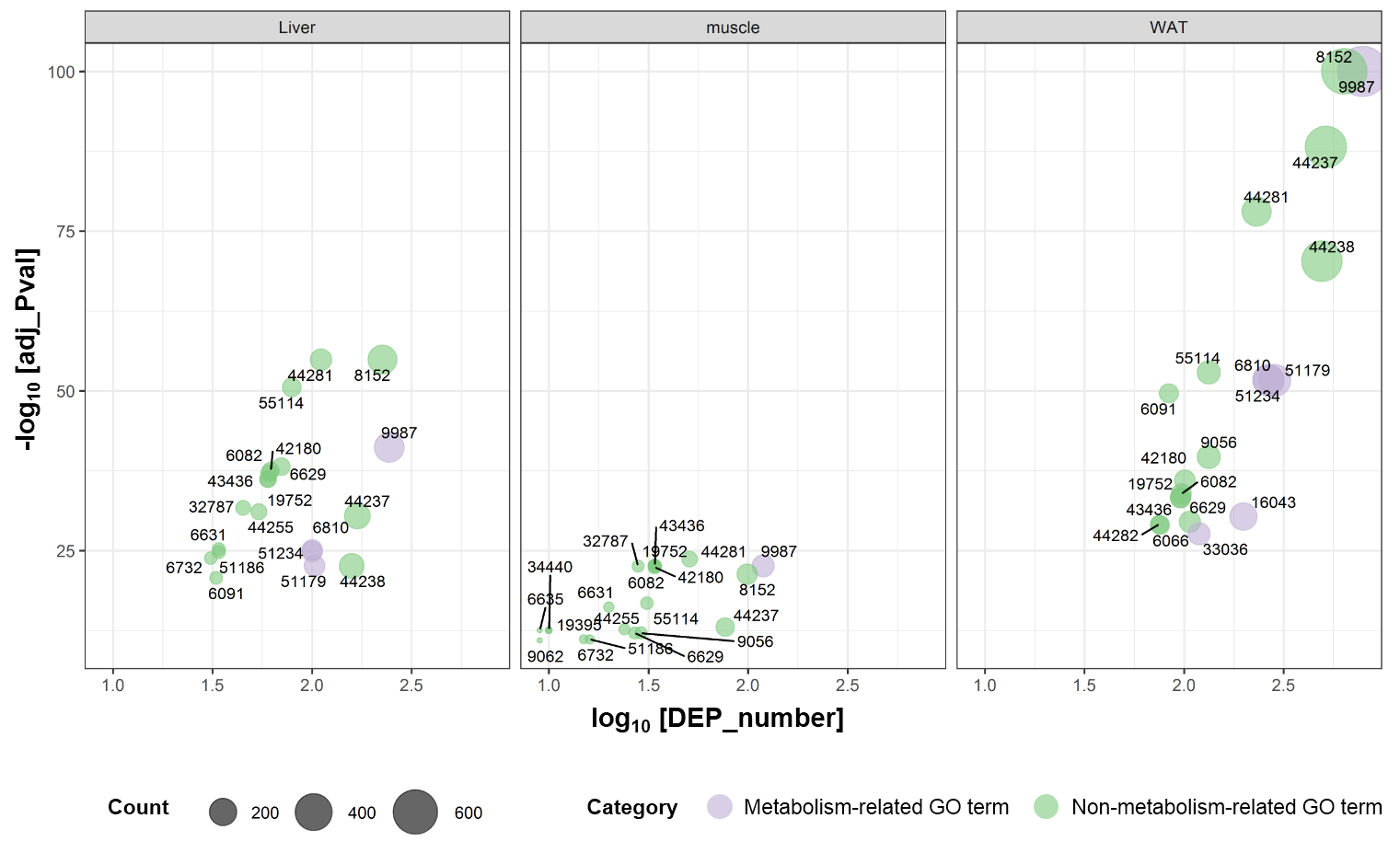


**Supplementary Figure 2. The** **TOP20 most enriched GO terms in WAT, Liver and skeletal muscle.** Where, GO terms were labeled by their GO IDs; metabolism-related GO terms were highlighted in violet and others were colored in green.


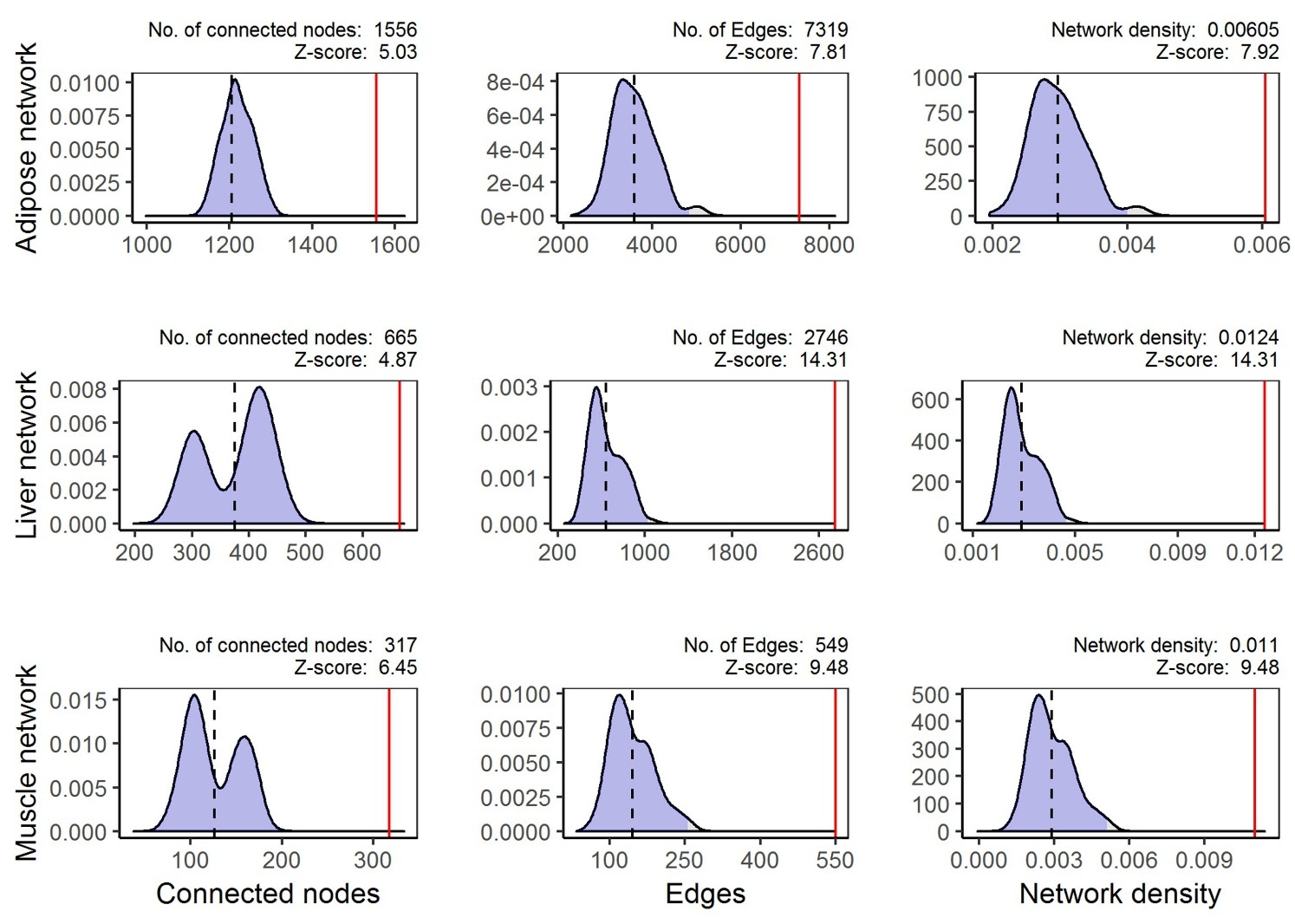


**Supplementary Figure 3. Distribution of connected nodes, edges and network density of 100 random networks.** Where, the distribution of global parameters was generated by kernel density estimation and the vertical lines colored in red represent the true value of these parameters in tissue-specific networks. Z-score > 2.33 denotes the parameter in tissue-specific network was significantly higher than random networks.
