# Supplementary material for: Exploring the Multi-Tissue Crosstalk Relevant to Insulin Resistance Through Network-Based Analysis
Source: Front Endocrinol (Lausanne). 2022 Jan 18;12:756785. doi: 10.3389/fendo.2021.756785 (PMC8805208; doi:10.3389/fendo.2021.756785)
Supplement: Supplementary file 1 [file DataSheet_1.zip › Supplementary Methods.DOCX]

Supplementary Methods

**TMT-based quantitative proteomics**

***Sample preparation.*** Total proteins were extracted from cell powder obtained by liquid nitrogen grinding of each sample. After that, four volumes of lysis buffer (8 M urea, 1% Protease Inhibitor Cocktail) was added to the cell powder, followed by sonication three times on ice using a high intensity ultrasonic processor (Scientz). The remaining debris was removed by centrifugation at 12,000 g at 4 °C for 10 min. Protein concentration was determined with BCA kit (P0011-1, Beyotime Biotechnology, Shanghai, China) according to the manufacturer’s instructions. For each tissue, equal protein extracts from every subject in IR and control group were separately pooled.

Protein solution was reduced with 5 mM dithiothreitol for 30 min at 56 °C and alkylated with 11 mM iodoacetamide for 15 min at room temperature in darkness. The protein sample was then diluted by adding 100 mM TEAB. Trypsin was added at 1:50 trypsin-to-protein mass ratio for the first digestion overnight and 1:100 trypsin-to-protein mass ratio for a second 4 h-digestion.

After trypsin digestion, Peptide was desalted by Strata X C18 SPE column (Phenomenex) and vacuum-dried. Peptide was reconstituted in 0.5 M TEAB and processed according to the manufacturer’s protocol for TMT kit (90068, ThermoFisher Scientific, Waltham, USA). The tryptic peptides were fractionated into fractions by high pH reverse-phase HPLC using Agilent 300Extend C18 column (5 μm particles, 4.6 mm ID, 250 mm length).

***LC-MS/MS analysis***. The tryptic peptides were dissolved in 0.1% formic acid (solvent A), directly loaded onto a home-made reversed-phase analytical column (15-cm length, 75 μm i.d.). The gradient was comprised of an increase from 6% to 23% solvent B (0.1% formic acid in 98% acetonitrile) over 26 min, 23% to 35% in 8 min and climbing to 80% in 3 min then holding at 80% for the last 3 min, all at a constant flow rate of 400 nL/min on an EASY-nLC 1000 UPLC system. The peptides were subjected to NSI source followed by tandem mass spectrometry (MS/MS) in Q ExactiveTM Plus (Thermo) coupled online to the UPLC. The electrospray voltage applied was 2.0 kV. The m/z scan range was 350 to 1800 for full scan, and intact peptides were detected in the Orbitrap at a resolution of 70,000. Peptides were then selected for MS/MS using NCE setting as 28 and the fragments were detected in the Orbitrap at a resolution of 17,500. A data-dependent procedure that alternated between one MS scan followed by 20 MS/MS scans with 15.0s dynamic exclusion. Automatic gain control (AGC) was set at 5E4. Fixed first mass was set as 100 m/z.

***Database search.*** The resulting MS/MS data were processed using Maxquant search engine (v.1.5.2.8). Tandem mass spectra were searched against SwissProt Mouse database concatenated with reverse decoy database (UniProt, 2019). Trypsin/P was specified as cleavage enzyme allowing up to 2 missing cleavages. The mass tolerance for precursor ions was set as 20 ppm in First search and 5 ppm in Main search. The mass tolerance for fragment ions was set as 0.02 Da. Carbamidomethyl on Cys was specified as fixed modification and oxidation on Met was specified as variable modifications. FDR was adjusted to < 1% and minimum score for peptides was set > 40.

UniProt, C. (2019). UniProt: a worldwide hub of protein knowledge. *Nucleic Acids Res* 47(D1)**,** D506-D515. doi: 10.1093/nar/gky1049.
